# Improving susceptibility of neuroendocrine tumors to radionuclide therapies: personalized approaches towards complementary treatments

**DOI:** 10.7150/thno.87345

**Published:** 2024-01-01

**Authors:** Susan Richter, Charlotte Steenblock, Alessa Fischer, Sandy Lemm, Christian G. Ziegler, Nicole Bechmann, Svenja Nölting, Jens Pietzsch, Martin Ullrich

**Affiliations:** 1 Institute for Clinical Chemistry and Laboratory Medicine, Faculty of Medicine and University Hospital Carl Gustav Carus, Technische Universität Dresden, Dresden, Germany.; 2 Department of Internal Medicine III, University Clinic Carl Gustav Carus, Technische Universität Dresden, Dresden, Germany.; 3 Department of Endocrinology, Diabetology and Clinical Nutrition, University Hospital Zurich (USZ), and University of Zurich (UZH), Zurich, Switzerland.; 4 Department of Radiopharmaceutical and Chemical Biology, Institute of Radiopharmaceutical Cancer Research, Helmholtz-Zentrum Dresden-Rossendorf (HZDR), Dresden, Germany.; 5 University Hospital Würzburg, Division of Endocrinology and Diabetes, Würzburg, Germany.; 6 Department of Medicine IV, University Hospital, Ludwig-Maximilians-University Munich, Munich, Germany.; 7 Faculty of Chemistry and Food Chemistry, School of Science, Technische Universität Dresden, Dresden, Germany.

## Abstract

Radionuclide therapies are an important tool for the management of patients with neuroendocrine neoplasms (NENs). Especially [^131^I]MIBG and [^177^Lu]Lu-DOTA-TATE are routinely used for the treatment of a subset of NENs, including pheochromocytomas, paragangliomas and gastroenteropancreatic tumors. Some patients suffering from other forms of NENs, such as medullary thyroid carcinoma or neuroblastoma, were shown to respond to radionuclide therapy; however, no general recommendations exist. Although [^131^I]MIBG and [^177^Lu]Lu-DOTA-TATE can delay disease progression and improve quality of life, complete remissions are achieved rarely. Hence, better individually tailored combination regimes are required. This review summarizes currently applied radionuclide therapies in the context of NENs and informs about recent advances in the development of theranostic agents that might enable targeting subgroups of NENs that previously did not respond to [^131^I]MIBG or [^177^Lu]Lu-DOTA-TATE. Moreover, molecular pathways involved in NEN tumorigenesis and progression that mediate features of radioresistance and are particularly related to the stemness of cancer cells are discussed. Pharmacological inhibition of such pathways might result in radiosensitization or general complementary antitumor effects in patients with certain genetic, transcriptomic, or metabolic characteristics. Finally, we provide an overview of approved targeted agents that might be beneficial in combination with radionuclide therapies in the context of a personalized molecular profiling approach.

## Introduction

Neuroendocrine neoplasms (NENs) are a heterogeneous group of tumors affecting neuroendocrine cells and arise in many different organs. NENs can occur in the digestive tract from enterochromaffin cells, the pancreas from islet or ductal cells, the lungs from pulmonary neuroendocrine cells, and the thyroid from parafollicular C-cells. Furthermore, pheochromocytomas originate from chromaffin cells of the adrenal medulla and paragangliomas from extra-adrenal ganglia (together referred to as PPGL).

Gastroenteropancreatic (GEP) NENs are divided into three grades based on proliferation markers. Grade 3 denotes fast-progressing tumors and is further divided into the group of poorly differentiated neuroendocrine carcinomas and well-differentiated neuroendocrine tumors, which are genetically unrelated and show different responses to chemotherapy 1. Some GEP-NENs are functionally active with various hormones, including serotonin, insulin, gastrin, glucagon, and vasoactive intestinal peptide, that can be produced and cause different clinical syndromes 2. About 27-45% of patients present with distant metastasis at first diagnosis 3, 4. Aggressiveness is generally dependent on the primary site, with NENs of the pancreas and small intestine having a high malignant potential but often indolent progression, and gastric and rectal NENs having lower rates of metastasis, but when metastases occur they progress rapidly. The liver is the most common site of metastasis in GEP-NENs. Currently used therapies are based on several successful phase III studies and include surgery (debulking or curative), local ablation of liver metastases, systemic therapy options with biotherapy using “cold” somatostatin analogs, PRRT, chemotherapy and molecularly targeted approaches 5.

Medullary thyroid carcinomas (MTCs) are a rare form of thyroid cancer with 5-10% of cases. MTCs often secrete calcitonin and can progress indolently after thyroidectomy due to their tendency of spreading to locoregional lymph nodes. Primary treatment is surgery of the thyroid gland and surrounding lymph nodes. Approaches for recurrent or metastatic MTCs include external beam radiotherapy, conventional chemotherapy, and tyrosine kinase inhibitors. Therapeutic strategies for metastatic MTC are rarely curative 6.

PPGL patients suffer from symptoms related to catecholamine excess, such as high blood pressure, sweating, headaches, and palpitations. The majority of PPGLs are curable by surgery, but up to 15% of pheochromocytoma and 40% of paraganglioma metastasize 7. Therapeutic options for patients with metastatic disease are limited and not curative. Currently recommended approaches include chemotherapy according to the Averbuch scheme for rapidly growing tumors, radiopharmaceutical therapy with iodine-131 meta-iodobenzylguanidine ([^131^I]MIBG) for slowly to moderately progressing tumors, and somatostatin receptor 2 (SSTR2) targeted peptide receptor radionuclide therapy (PRRT) 8, 9.

The following sections summarize clinically approved radionuclide therapies for NENs, review molecular determinants of radioresistance in the light of what is known about cancer stem cells in NENs, and discuss strategies for improvement using adjuvant radiosensitizers with the potential to enhance treatment efficacy and reduce therapeutic escape.

## Clinically Approved Radionuclide Theranostics in NENs

### Somatostatin type 2 receptor targeting

**Figure 1:**
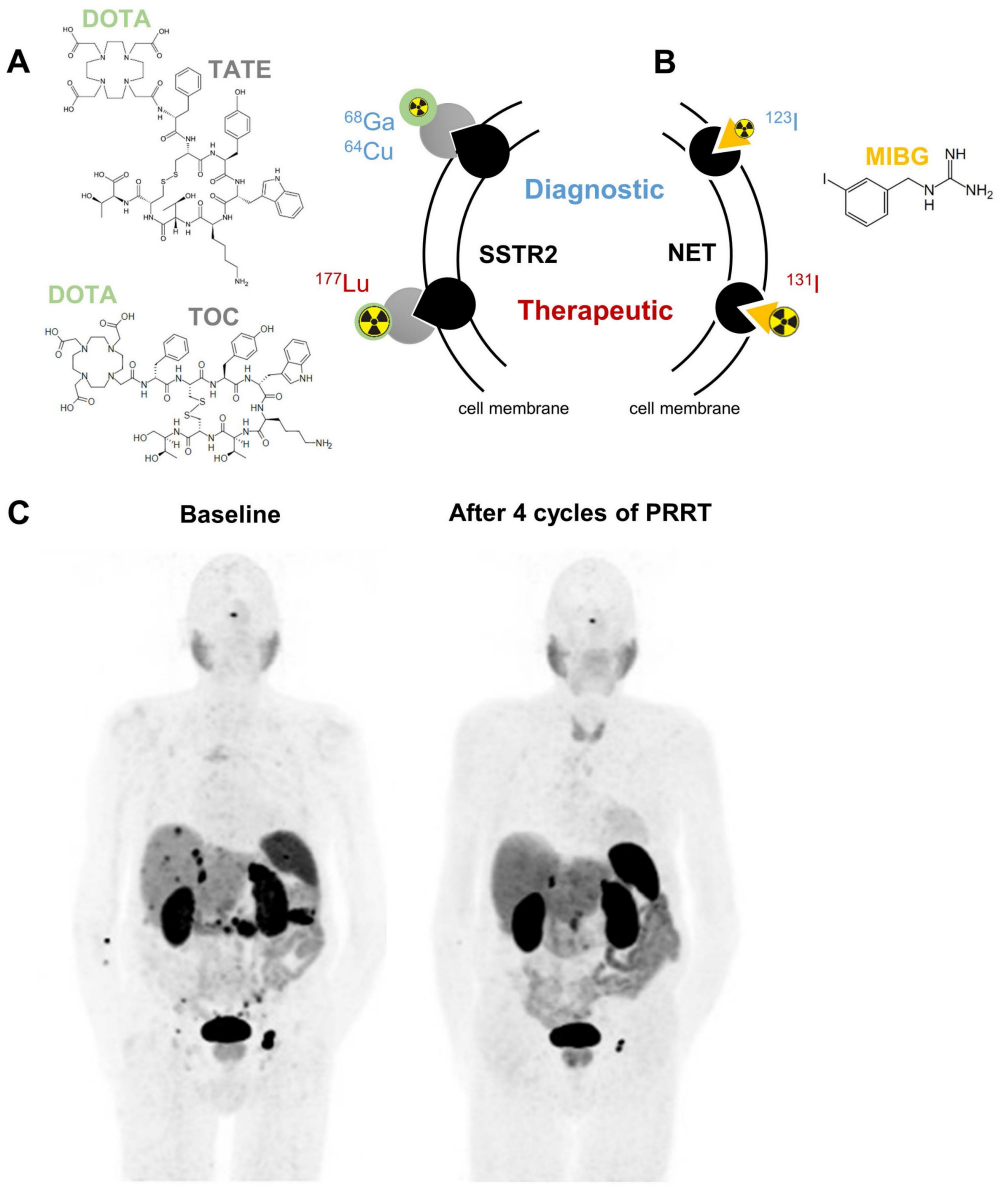
Clinically approved targeted radionuclide theranostics in NENs mediated by somatostatin receptor type 2 (SSTR2, A) or norepinephrine transporter (NET, B). Panel C shows representative ^68^Ga-DOTATATE PET/CT images of an 83-year old male patient presenting with a well-differentiated neuroendocrine tumor of the pancreas with peritoneal, lymph node and hepatic metastases at baseline. After 4 cycles of PRRT, a good partial response with shrinkage of the pancreatic tumor, peritoneal metastases and the lymph node metastasis inguinal left was observed. Abbreviations: DOTA - tetraxetan, TATE - (Tyr3)octreotate, TOC - (Tyr3)octreotide, MIBG - meta-iodobenzylguanidine

Somatostatin receptors belong to the family of G-protein coupled receptors; they regulate hormone secretion and cell proliferation. Especially subtype 2 (SSTR2) is strongly expressed in NENs exceeding levels of healthy tissues 10, 11. This tumor selectivity can be targeted using radiolabeled somatostatin analogs 12. Depending on the radionuclide attached, these agents are valuable for diagnosis by positron emission tomography (PET) imaging or for radionuclide therapy. To express these dual benefits the term theranostics is used (Figure 1).

Preoperative PET imaging with the positron (β^+^)-emitting somatostatin analog [^68^Ga]Ga-DOTA-(Tyr^3^)octreotate (TATE) is recommended in the guidelines for NENs and was FDA (Food and Drug Administration)-approved in 2016 in the Unites States 13-16. [^68^Ga]Ga-DOTA-TATE detects primary and metastatic lesions of NENs with high sensitivity and specificity of over 80%, as demonstrated in multiple studies 17-19. Furthermore, [^68^Ga]Ga-DOTA-TOC and [^64^Cu]Cu-DOTA-TATE are available as FDA-approved diagnostic radiopharmaceuticals 20, 21. Beta-minus (β^-^) particle-emitting SSTR2-binding somatostatin analogs are used for therapeutic intervention in GEP-NENs and PPGLs (Figure 1A/C) 12, 22. In 2018, PRRT with [^177^Lu]Lu-DOTA-TATE was FDA approved as a promising treatment modality for patients with well-differentiated metastatic/inoperable GEP-NENs due to the NETTER-1 phase III trial that reported improved progression-free survival and quality-of-life in patients with progressing midgut NENs 23. Disease was controlled in 82% of patients with progression-free survival of 29 months and overall survival of 63 months 24, 25. Also poorly differentiated grade 3 GEP-NENs benefit from PRRT as long as increased uptake of [^68^Ga]Ga-DOTA-TATE is confirmed in all lesions 26.

MTCs express SSTR2, which positively correlates with improved overall survival 27, 28. Imaging analysis with [^68^Ga]Ga-DOTA-TATE was reported to be especially positive in patients with high serum calcitonin, indicating that more differentiated MTCs have higher expression levels of SSTR2 29, 30. In accordance, [^177^Lu]Lu-DOTA-TATE therapy was recently shown to achieve favorable responses in patients with metastatic MTC 31.

In PPGLs, SSTR2 is expressed in about 50% of tumors depending on the underlying mutation 11, 32. PPGLs that arise due to loss-of-function mutations in succinate dehydrogenase (SDH) subunit genes show excellent detection rates with [^68^Ga]Ga-DOTA-TATE, whereas PPGLs with gain-of-function mutations in hypoxia-inducible factor 2α (HIF2α or EPAS1) are less well detectable 33, 34. Several clinical studies suggest that PRRT is one of the most effective therapies for metastatic PPGLs, especially for those with *SDHx* mutations 7, 35. A recently published meta-analysis estimated the disease control rate in PPGLs treated with PRRT at 81% 36. PRRT is well tolerated with limited acute and medium-term toxicity profiles and only low rates of nephrotoxicity and therapy-related myeloid neoplasms. Therefore, PRRT is a viable option for clinicians to delay tumor progression in patients with metastatic PPGL. Most patients show at least a partial response with progression-free survival in the range of 14 to 39 months and a median overall survival of 50 months 37, 38. Unfortunately, many patients treated with PRRT will progress eventually and a recent report describes two patients with extensive metastatic spread after initial response to [^177^Lu]Lu-DOTA-TATE 39. An ongoing phase II trial (NCT03206060) is evaluating [^177^Lu]Lu-DOTA-TATE in metastatic and inoperable PPGL.

### Norepinephrine transporter targeting

Another molecular target for theranostic radiopharmaceuticals is the norepinephrine transporter (encoded by *SLC6A2*). It is mainly expressed in noradrenergic neurons and chromaffin cells of the adrenal medulla and facilitates norepinephrine uptake. The norepinephrine transporter substrate meta-iodobenzylguanidine (MIBG) labeled with suitable radioisotopes of iodine is used for imaging (mainly [^123^I]MIBG) and therapy ([^131^I]MIBG) (Figure 1B). Planar scintigraphy or single-photon emission computed tomography (SPECT) are used as standard imaging techniques for these gamma-photon (γ)-emitting radiopharmaceuticals. Uptake of [^123/131^I]MIBG was demonstrated in all types of NENs, but predominantly in PPGLs and neuroblastomas of the adrenal 40.

For diagnostic use, low-dose [^131^I]MIBG was already approved in 1994 by the FDA as an imaging agent for the localization of PPGL, later augmented by [^123^I]MIBG in 2008. Since 2018 the formulation of high-specific-activity (HSA) [^131^I]MIBG, which contains only a negligible amount of unlabeled MIBG, is currently the only FDA-approved therapy for metastatic PPGLs in the United States. Therapeutic benefits include sustained blood pressure control with partial response or stable disease over 12 months in over 90% of patients; median overall survival was 36.7 months 41. Generally, a more differentiated tumor phenotype with a mature storage and secretion apparatus for catecholamines or other hormones, such as serotonin, are favorable for MIBG uptake 42. For this reason, a currently running phase II study in Brazil is recruiting patients with well-differentiated NENs for [^131^I]I-MIBG therapy (NCT04831567). Further details on [^131^I]MIBG therapy in different NENs have been summarized elsewhere 43. Especially for PPGLs, there might be cases with positive imaging results for [^131^I]MIBG and [^68^Ga]Ga-DOTA-TATE, making the patient eligible to both kinds of targeted radionuclide therapy. Practical recommendations for decision-making in such instances were recently published 44.

Besides FDA-approved radiopharmaceuticals for theranostic targeting of SSTR2 and norepinephrine transporters in NENs, a broad range of theranostic agents using other radionuclides or directed towards other tumor-specific molecular targets are currently under preclinical and clinical evaluation. This topic was extensively reviewed elsewhere 45-47.

### Biophysical and radiobiological effects of beta-minus particle-emitting radiopharmaceuticals

Most of our current knowledge on radiobiological effects in cancer emerged from studies using external beam radiation therapy (EBRT). However, the biophysical and radiobiological effects of EBRT differ significantly from those of radionuclide therapy. Unlike EBRT, the effects of radionuclide therapy depend not only on the type of radiation applied at a specific dose, but are also considerably influenced by the distribution and retention of the radiopharmaceutical within the target tissue, the half-life of the radionuclide, and the varying ionization density of the emitted particles.

To date, targeted radionuclide-based therapies of solid tumors, including NENs, are carried out mainly with β^-^ particle emitters. Despite their rather low *in vitro* cytotoxicity compared to α particle or Auger electron emitters, these radionuclides continue to be pursued for targeted therapies, mainly due to their availability and favorable physical characteristics, such as particle energy and range (leading to crossfire irradiation of tumor cells with no radiopharmaceutical bound) and physical half-lives compatible with the biological half-lives of the carrier molecules 48. Nevertheless, especially non-uniformities in the activity distribution as well as declining activity concentration over time will usually result in sub-lethal low-dose irradiation at least in some regions of the target tissue, where the tumor cells will escape from treatment or eventually develop a radioresistant phenotype. Crucial factors contributing to a non-uniform radiopharmaceutical distribution in solid tumors are (i) differences in perfusion, e.g. depending on the amount of connective tissue and/or vascularization density, (ii) region-specific differences in interstitial pressure, and (iii) differences in binding-site densities among tumor cells.

**Figure 2:**
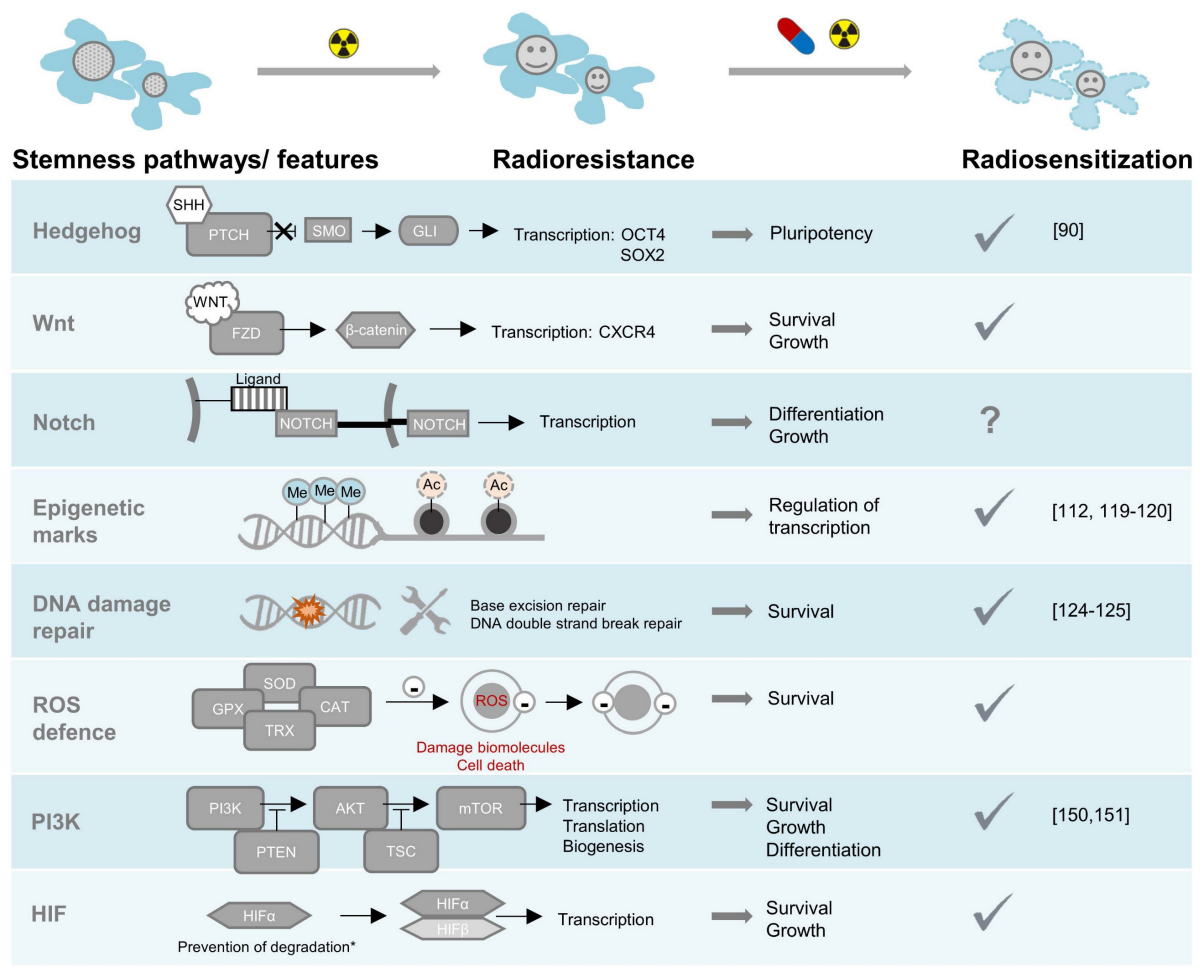
Radioresistance pathways in NENs: pharmacological targeting sensitizes to radiotherapy. A tick under radiosensitization indicates that preclinical evidence for radiosensitization exists, the citations specify that evidence was given specifically in NEN models. *HIFα degradation can be perturbed by pathogenic variants in several genes or through metabolic effects of oncometabolites. Abbreviations: SHH-sonic hedgehog, PTCH-patched, SMO-smoothened, GLI-glioma-associated oncogene, FZD-frizzled, Me-methylation, Ac-acetylation, ROS-reactive oxygen species, SOD-superoxide dismutase, CAT-catalase, GPX-glutathione peroxidase, TRX-thioredoxin, PI3K-phosphatidylinositol 3-kinases, AKT-serine/threonine-protein kinase, PTEN-phosphatase and tensin homolog, TSC-tuberous sclerosis complex, mTOR-mammalian target of rapamycin, HIF-hypoxia inducible factor.

There are two main strategies for enhancing the efficacy of conventional radionuclide therapies: increasing the absorbed radiation dose (dose maximization), or enhancing the tumor's susceptibility to the biophysical and radiobiological effects of ionizing radiation (radiosensitization). The absorbed dose of a target-specific radiopharmaceutical can be improved by (i) optimizing treatment schemes and sequences, e.g., via personalized fractionating and dosing 49; (ii) minimizing off-target retention, in particular in kidneys, thereby allowing for increased total applied doses of the radiopharmaceutical 16, 50, 51; (iii) upregulating molecular targets for radiopharmaceuticals in tumors, e.g., through epigenetic modifiers 52-55; (iv) increasing the bound fraction of radiopharmaceuticals in tumors, e.g., using albumin binder conjugates providing increased blood circulation times 56 or receptor antagonists 57, 58; and (v) attaching alternative radionuclides with suitable chemical properties for radiolabeling as well as favorable decay properties providing radiation with high ionization densities such as α particles, Auger electrons and/or high-energy β^‒^ particles, and half-lives compatible with the pharmacologic properties of the targeting vectors, e.g. ^225^Ac, ^213^Bi, ^211^At, ^212^Pb, ^161^Tb, and ^67^Cu 59-61. Such dose maximization approaches were reported elsewhere and will only be discussed in respect to modulation of *SSTR2* expression. The following sections outline pathways causing radioresistance in NENs and highlight concepts for combining clinically approved radionuclide therapy with targeted small molecules to achieve radiosensitizing effects (Figure 2).

## Strategies for Enhancing Susceptibility to Conventional Radionuclide Therapies: Molecular Targets in NENs

### Cancer stem cell-like traits in NENs

Radioresistance is closely linked to stem cell traits, i.e. the ability of self-renewal. Reports from different tumor entities have shown that so called cancer stem cells (CSCs) are inherently resistant to radiation treatment through different mechanisms, including enhanced DNA repair capabilities, ROS detoxification strategies, and activation of cell survival pathways, such as PI3K/Akt, Wnt/β-catenin, and Notch signaling 62. Here we review what is known about CSCs in NENs.

CSCs have been identified in gastrointestinal NENs based on aldehyde dehydrogenase (ALDH) activity, a marker known to promote self-renewal 63. ALDH-positive cells showed upregulated signaling of tyrosine-protein kinase Src, which was important for tumor growth in a xenograft model. In pancreatic NENs, a population of CD90 and ALDHA1 expressing cells was found to be highly tumorigenic and characterized by C-Met signaling, another tyrosine-protein kinase 64. As an additional marker for these stem-like cells, CD47 was identified. Targeting CD47 increased tumor cell destruction by macrophages and resulted in reduced tumor growth and prolonged survival in mice. Additional anti-EGFR therapy increased survival even further. In MTC, a CD133-positive cell population was found to be enriched in tumor spheres 65. Additionally, the authors showed sphere formation to be dependent on the receptor tyrosine kinase Ret. CD133-positive cells were also involved in chemoresistance to 5-fluorouracil 66. A study identified co-expression of the stem cell markers CD133 and CD44 to be correlated with invasion and metastases and decreased patient survival 67. CD133-positive cells and other stem cell markers were also identified in small cell lung cancer (SCLC) in multiple studies 68-70. A side population of cells with high proliferation rates, efficient tumor-inducing ability, higher angiogenic potential, and decreased neuronal markers of CD56 and CD90, was identified in different SCLC cell lines 71. These cells expressed high levels of genes associated with cancer stem cells, Notch and Hedgehog pathways. Both pathways are involved in the regulation of stem cell features.

In addition to residing CSCs being the cause of tumor development and malignant transformation, there are indications that populations of dedifferentiated cancer cells contribute to tumor progression and invasiveness, in which mutations cause the acquisition of more stem-like cell features. This hypothesis in the context of NENs was outlined in a recent review 72. Specific experimental evidence for either theory is, however, scarce for these tumor entities. Intra-tumor heterogeneity caused through genome instability and the occurrence of cells with different mutational changes can drive progressive dedifferentiation of tumors and varies considerably between different types of NENs. High numbers of subclonal cell populations were identified in pancreatic NENs, e.g. with* MEN1* mutations as subclonal drivers 73. Additionally, different methylation signatures corresponding to genomic subgroups have been described in pancreatic NENs, in which the group with the highest methylation pattern is enriched for *MEN1* mutations 74. Pancreas-specific knockout of *Men1* in mice was shown to result in insulinomas with progressive loss of insulin production, indicating dedifferentiation processes 75. In contrast, PPGLs have low numbers of subclonal populations and a low mutational burden compared to other tumors 76, 77. A model has been proposed for PPGLs, in which tumors with more immature features, e.g. those due to *SDHx* mutations, arise from earlier progenitors, like Schwann cell precursors and carry more stem-like features, while others originate from mutations in later developmental stages 78.

Radiation and chemotherapy can also enrich and transform more differentiated cells into stem-like cells resulting in relapse or therapy resistance. These cell populations are characterized by a transition to a mesenchymal state; they have increased survival, DNA damage repair capacity, and activated Hedgehog, Wnt, Notch, HIF, and phosphoinositide 3-kinase (PI3K) signaling 79.

### Hedgehog signaling

Several mechanisms can lead to activation of Hedgehog signaling, mutations of pathway components, such as loss-of-function of the Smoothened (SMO) repressor *PTCH1,* or constitutive activation of SMO, excessive expression of Hedgehog ligands either by the tumor cell itself or by stromal components 80. One of the transcriptional targets of Hedgehog signaling is NANOG, known as a master transcription factor regulating the recruitment of other transcription factors, including those involved in pluripotency and self-renewal, e.g. OCT4 and SOX2 81, 82. Several studies have shown that the growth of SCLC cells is strongly dependent on Hedgehog signaling 83, 84. The majority of ileum NENs express the ligand *SHH*, other components of the Hedgehog pathway are also expressed in gastrointestinal NENs 85, 86. Additionally, a subgroup of PPGLs with *MAML3* fusion or CSDE1 somatic variants is characterized by activation of Hedgehog and Wnt signaling 87. Although specific information for NENs is limited, currently available studies indicate that Hedgehog plays an important role in these tumors. Whether the activation of Hedgehog signaling only occurs in CSCs remains to be elucidated, but at least it suggests a poorly differentiated phenotype, which in turn might lead to increased invasiveness and therapy resistance, e.g., against radiation treatment 88, 89. In fact, the Hedgehog inhibitor sonidegib was shown to potentiate peptide receptor radiotherapy in a NEN cell line 90. Sonidegib is FDA-approved for advanced basal cell carcinoma. Severe side effects can occur with high doses, but lower doses achieved better benefit-to-risk profiles 91.

### Canonical Wnt signaling

Wnt proteins activate transmembrane receptors of the Frizzled family, and downstream actions prevent phosphorylation of β-catenin, which in turn accumulates and forms transcription-initiating complexes. Inhibition of this pathway demonstrated reduced viability, growth and colony-forming ability in NEN cell line models 92, 93. Furthermore, the promoter of the Wnt signaling inhibitor secreted frizzled-related protein 1 (SFRP-1) was found to be methylated in many NEN tissues and cell line models 93. Treatment of cell lines with DNA methylation inhibitor 5-aza-2′-deoxycytidine increased expression of SFRP-1 and other negative regulators of Wnt. Pathway inhibitors were shown to have radiosensitizing effects in other cancer entities 94. Wnt signaling was also identified to regulate CXCR4 expression, indicating that NENs with Wnt signaling activation might benefit from [^68^Ga]Ga-pentixafor and [^177^Lu]Lu-/[^90^Y]Y-pentixather theranostics rather than SSTR2 targeting 95. Especially pancreatic NENs with *MEN1* mutations show activated Wnt signaling and could be susceptible to this form of theranostics 96.

### Notch signaling

Notch is another evolutionarily conserved pathway that regulates proliferation, stem cell features and differentiation. Notch ligands bind to their receptors and activate canonical signaling through the transcription factor CSL (CBF-1/Suppressor of Hairless/LAG1); alternatively, non-canonical signaling independently of CSL can occur, e.g., through antagonistic interaction with Wnt/β-catenin 97. Notch signaling was shown to be a regulator of neuroendocrine differentiation in pancreatic, lung, thyroid, and gastrointestinal tissues and NENs 98-102. Moreover, head and neck paragangliomas were shown to have amplifications in Notch pathway components 103. NOTCH1 and its receptor were also expressed in paragangliomas without copy number variations through a possible mechanism involving miRNA. Nevertheless, activation of NOTCH1 through epigenetic modulation in NENs was associated with treatment response in cell line models and patients 104, 105, emphasizing the importance of careful target evaluation in each disease setting or even patient.

### Epigenetic reprogramming

Epigenetic gene signatures, including DNA methylation and histone methylation, and acetylation, play an important role in maintaining stemness. Alterations in epigenetic marks during carcinogenesis affect cellular plasticity and reprogramming. Methylation signatures corresponding to different subtypes were found in pancreatic NENs 74. Additionally, mutations in genes involved in epigenetic processes, including histone modification, were identified 106. Several histone deacetylases (HDACs) were elevated in high-grade (G3) pancreatic NENs compared to lower grades. Especially upregulation of nuclear HDAC5 was associated with reduced disease-free survival and overall survival 107. The non-selective HDAC inhibitor panobinostat showed promising results *in vitro* and stimulated redifferentation evaluated by insulin production and *SSTR2* expression 108. The latter effect is of particular interest, since increased presence of the target molecule SSTR2 is likely to improve efficacy. Several preclinical studies evaluated the effects of DNA methyltransferase and HDAC inhibitors in GEP-NEN cell lines for its ability to increase SSTR2 109-111. Combination of 5-fluorouracil with either decitabine (DNA methyltransferase inhibitor) or tacedinaline (HDAC inhibitor) increased cell apoptosis compared to either drug alone, had radiosensitization effects when given before gamma irradiation, and increased SSTR2 in several human NEN cell lines 112. As a result, cellular uptake of [^68^Ga]Ga -DOTA-TOC was higher with the drug combination. Although SSTR2 upregulating effects have been shown *in vitro* and *in vivo*, the underlying mechanism may be unrelated to epigenetic reprogramming 111. To this end, our group showed that epigenetic modification with valproic acid and 5-aza-2'-deoxycytidine modulates SSTR2 levels and sensitivity to [^177^Lu]Lu-DOTA-TATE in mouse models for PPGL 53. Similar to experiences in GEP-NEN models, *SSTR2* promoter methylation was also not the cause of SSTR2 induction in PPGL.

In PPGLs, mutations in genes encoding Krebs cycle enzymes, such as SDH or fumarate hydratase (FH), lead to a CpG island methylator phenotype (CIMP) associated with transition to a mesenchymal state and increased malignancy due to inhibition of ten-eleven translocation (TET) methylcytosine dioxygenases by oncometabolites 113. Importantly, it was demonstrated that the CIMP only leads to metastatic features in conjunction with HIF2α activation in PPGL 114, 115. This suggests that epigenetic targeting alone may not be successful, but might be worth investigating in combination and in patients with rare germline pathogenic variants in DNA methyltransferases 116.

Heat shock protein 90 (HSP90) is a molecular chaperone that binds to chromatin regulators, is highly expressed in NEN primary tumors and metastases, and targeting was shown to have antiproliferative effects 117, 118. Combination treatment of [^177^Lu]Lu-DOTA-TATE with HSP90 inhibitor onalespib in a NEN xenograft model increased tumor doubling time, the percentage of cases with complete remissions, and overall survival compared to control or monotherapy 119, 120. Another study investigated CC-90011, an oral inhibitor of lysine-specific demethylase 1 (LSD1/KDM1A) in a phase I trial 121. Monotherapy was well-tolerated in patients with refractory NENs, and four patients achieved prolonged stable disease (longer than 6 months). In addition, proteins reading histone modifications have been targeted in NENs. Inhibitors for bromodomain-containing proteins from the bromo and extra-terminal (BET) domain family inhibited proliferation and increased apoptosis in preclinical NEN models 122. Currently, one trial tests [^177^Lu]Lu-DOTA-TATE together with DNA hypomethylating agent ASTX727 (NCT05178693).

### DNA damage repair

Enhanced DNA repair capacity protects genome integrity and is required for maintaining cell stemness. Hence, it is not surprising that also CSCs are characterized by enhanced DNA damage response and/or elongated cycling times through activation of cyclin-dependent kinases, which in turn leads to more time for DNA repair processes 123. Inhibiting the DNA damage capacity in tumors is a valid strategy. Preclinical studies in SSTR2-expressing neuroendocrine cell lines or tumor slices demonstrated that poly(ADP-ribose) polymerase-1 (PARP) inhibitors talazoparib, olaparib, and 1,5-dihydroxyisochinolin in combination with [^177^Lu]Lu-DOTA-TATE increased the number of double-strand breaks and reduced cell/tumor growth further than either treatment alone 124, 125. In a PPGL cell line model with *SDHB* knockdown, the combination of olaparib with temozolomide resulted in reduced metastatic lesions and prolonged overall survival in mice 126. Several clinical studies are underway to further investigate safety and efficacy of combining PARP inhibitors with [^177^Lu]Lu-DOTA-TATE (NCT04375267, NCT04086485, NCT05053854, NCT05870423). At least in combination with EBRT, olaparib demonstrated an excellent toxicity profile 127. Further radiosensitizing agents that are currently tested in clinical trials include Triapine, an inhibitor of ribonucleotide reductase, which is the rate-limiting enzyme of DNA synthesis and repair (NCT05724108), and the DNA-dependent protein kinase inhibitor Peposertib (NCT04750954).

### Reactive oxygen species (ROS) and detoxification mechanisms

Ionizing radiation triggers ROS in cells through radiolysis of water and the induction of ROS formation in mitochondria 128. Excessive ROS in turn damages biomolecules and activates different cell death pathways 129. Consequently, the efficacy of radiation treatment is linked to a cell's ability to control ROS levels through various detoxification mechanisms. These include enzymes like manganese or copper-zinc superoxide dismutase, catalases, and glutathione peroxidase (GPX)/reductase that convert oxygen radicals to hydrogen peroxide and subsequently water, and nonenzymatic antioxidants, such as urate, glutathione, polyamines, vitamins E, A, and C. Tight regulation of ROS levels is also important for maintaining stemness; hence all pathways described above influence redox homeostasis 130. GPX4 is one such regulator that protects cells from lipid oxidation and thereby protects from ferroptotic cell death 131. SCLC cell lines with a non-neuroendocrine phenotype were more sensitive to ferroptosis induction by GPX4 inhibition than SCLC cell lines with a more neuroendocrine subtype, which relied more on the thioredoxin antioxidant pathway 132. Both pathways have been implicated in radiosensitization of cancer cells 133, 134. Another important regulator of redox homeostasis is the nuclear factor erythroid 2-related factor 2 (NRF2). It induces the expression of a multitude of antioxidant enzymes and metabolic components participating in the pentose phosphate pathway, which generates NADPH necessary for the regeneration of glutathione. In a model for PPGL with low *SDHB* expression the NRF2 inhibitor brusatol showed cytotoxic effects 135. It was also demonstrated to enhance radiosensitivity in other cancer cells 136.

### PI3K signaling

The PI3K pathway with its downstream regulators, the serine/threonine kinase AKT and mammalian target of rapamycin (mTOR), has been implicated in the development and progression of many cancers due to its role in mediating cell survival and proliferation. Furthermore, this pathway can be activated in response to radiation treatment, thereby facilitating radioresistance. Current knowledge was gathered mainly through studies in more common cancers, such as glioblastoma, non-small cell lung cancer, head and neck cancer, colorectal cancer, and prostate cancer, and is summarized in a recent review 137. Interestingly, the use of single inhibitors of pathway components generated variable results, while dual PI3K/mTOR inhibitors demonstrated more consistent radiosensitization effects that were associated with inhibition of double strand break repair and cell proliferation, and elevation of apoptosis and autophagy. Additionally, effects on the tumor microenvironment contributed to radiosensitization by normalizing the tumor vasculature and thereby reducing areas of low oxygen.

Different mechanisms were shown to result in PI3K/AKT/mTOR activation in GEP-NENs, including gene mutations in pathway components, copy number gain of AKT1 or AKT2, decreased expression of mTOR regulators PTEN and TSC2 or increased expression of tumorigenic pathway components 106, 138-140. PPGLs are classified based on their expression profile in clusters. Especially tumors grouped into cluster 2 are characterized by PI3K/AKT/mTOR activation due to mutations in the RET proto-oncogene (*RET*), neurofibromin 1 (*NF1*), transmembrane protein 127 (*TMEM127*), MYC-associated factor X (*MAX*), and other related genes 141, 142. Mutations in RET, both germline and somatic, also cause the development of MTC, where activated PI3K/AKT/mTOR signaling was demonstrated in primary tumors and metastasis 143, 144.

Inhibition of the PI3K/AKT/mTOR pathway demonstrated anti-tumor effects in several models of GEP-NENs, MTC, and PPGL 145-149. In one study a link between anti-tumor activity and the induction of differentiation was demonstrated in GEP-NEN cell lines 149. Additionally, radiosensitizing effects of PI3K/AKT/mTOR inhibitors have been reported in *in vitro* models of GEP-NENs 150, 151. In PPGLs with known metastatic progression, a microRNA signature was identified that functions as a potential marker for tumors with higher sensitivity to mTOR pathway inhibition 152. Three trials are registered that investigate the combination of tyrosine kinase or mTOR inhibitors with [^177^Lu]Lu-DOTA-TATE (NCT05687123, NCT05249114, NCT03629847). A previous study found that everolimus caused manageable toxicity as a second agent to [^177^Lu]Lu-DOTA-TATE 153.

### HIF signaling

Another important pathway in NENs that is associated with PI3K/AKT/mTOR signaling is mediated through the transcription factors HIF1α and HIF2α. The latter appears to be more relevant for NEN development and progression, since HIF2α is a regulator of trunk neural crest stemness and migration towards sympathoadrenal sites 154.

PPGLs of the expression cluster 1 arise due to mutations in genes activating hypoxia-inducible factors (HIFs), but especially HIF2α, and are more prone to metastasize 115. Genes conferring susceptibility for cluster 1 PPGLs also include SDH subunit genes and *FH* that result in production of the oncometabolites succinate or fumarate, which inhibit α-ketoglutarate-dependent enzymes and thereby induce HIF signaling and global epigenetic modifications 113, 114. These metabolic features can be used to identify such tumors, also those where succinate increases are not caused by gene mutations but rather by epigenetic mechanisms 155, 156. Cluster 1 PPGLs most likely arise from less differentiated chromaffin cells than cluster 2 PPGLs, indicated by the more immature nature of the catecholamine production and secretion machinery and the earlier age of disease onset 78.

In PPGL cell models, HIF2α activation is associated with increased migratory and invasive features, a less differentiated cellular state, and a more radioresistant phenotype 114, 115, 157. Radiosensitizing properties have been described for SN-38 through the inhibition of radiation-induced HIF1α in colorectal cancer 158; however, whether HIF2α inhibition can lead to similar effects in NENs has to be explored. The specific HIF2α inhibitor belzutifan is currently tested as a monotherapy in a phase II clinical trial in patients with pancreatic NENs and PPGL (NCT04924075). Preclinical evidence whether belzutifan can act as a radiosensitizer in combination with ^177^Lu]Lu-DOTA-TATE is, unfortunately, still outstanding.

HIF-regulated processes open up further possibilities for targeted radiosensitizing approaches. Exemplarily, an attractive molecular target in the specific context is cyclooxygenase-2 (COX-2) and subsequent prostaglandin-mediated signaling pathways 159. Prostaglandins produced due to increased COX-2 activity, such as PGE_2_, are pro-inflammatory cytokine-like factors that substantially modulate the immune response in cancer. They are also involved in the establishment of the tumor-associated microenvironment by promoting angiogenesis. In this regard, prostaglandins are directly involved in tumor progression and the acquisition of radioresistance, which leads to unsatisfactory results of radiotherapy 160. Additionally, the role of COX-2 in CSC survival and recolonization after therapy has been considered in detail elsewhere, making it clear that inhibiting COX-2 is an effective way to prevent treatment failure due to tumor repopulation 161. Addressing COX-2 by selective inhibitors or dual drugs as an adjuvant approach is also feasible in NENs 162-165.

### Metabolic interventions

The acquisition of proliferative, malignant, and stem cell features during tumor progression is accompanied by changes in cell metabolism, raising the possibility of targeting deregulated metabolic features. Highly glycolytic tumors, evident from strong uptake of [¹⁸F] fluorodeoxyglucose (FDG) by PET, could benefit from radiosensitization with glycolytic inhibitors. NENs double positive for [^68^Ga]Ga-DOTA-TATE and [¹⁸F]-FDG have been observed previously 166. On the other hand, targeting mitochondrial metabolism reduces oxygen usage and can result in elevated generation of reactive oxygen species. Both approaches are still experimental. Radiosensitizing effects of glycolytic inhibitor 2-deoxy-D-glucose and respiratory complex I inhibitor metformin have been demonstrated in neuroblastoma, glioma and other cell lines in combination with ionizing radiation 167. Additionally, retrospective analysis of patients taking metformin as an anti-diabetic drug during radiotherapy suggests potentiating effects 168. Interestingly, CSCs either exhibit a glycolytic or mitochondrial phenotype depending on the tissue of origin 169. Further research is required to understand these mechanisms in NENs. Targeting of other metabolic pathways may also be beneficial in certain subgroups of NENs. PPGLs with defects in *SDHx* genes show increased dependency on glutamine metabolism for replenishing Krebs cycle intermediates 170. The feasibility of targeting glutamine metabolism in combination with radiation to enhance radioresponse was recently demonstrated in prostate cancer cell lines 171. Furthermore, preclinical evidence suggests that pharmacological inhibition of nicotineamide phosphoribosyltransferase (NAMPT), an enzyme involved in NAD^+^ metabolism, sensitizes the NEN cell line GOT1 to [^177^Lu]Lu-DOTA-TATE 172. NAMPT is responsible for regeneration of NAD^+^, which is required amongst others for activation of PARP-1, thereby indirectly influencing DNA repair.

## Personalized radiosensitization strategies for SSTR2-based PRRT

Although many promising approaches towards improving SSTR2-based PRRT in NENs have been demonstrated in preclinical models, for patients it will be important to select the potentially most effective radiosensitization agent on an individual basis. Patients should be characterized based on germline mutational status, as well as the mutational, transcriptomic, and metabolomic profile of the primary tumor. Additionally, biopsies of metastases or inoperable tumors could be analyzed where possible.

**Figure 3:**
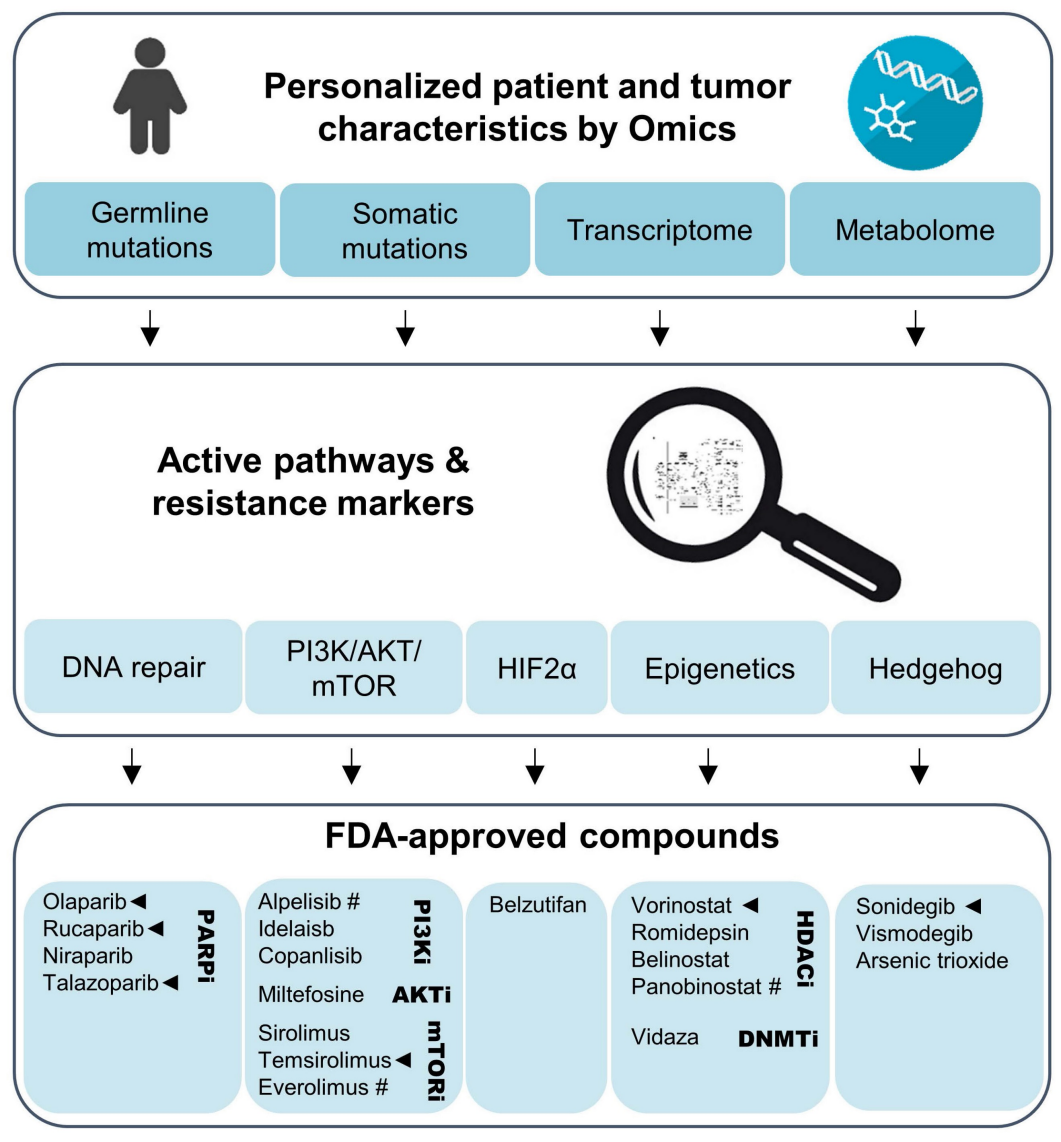
Personalized evaluation of promising radiosensitizers for combination therapy with PRRT in NENs. Omics studies on gene mutations, transcriptional and metabolic pathways in the primary tumor or biopsy material should lead the selection of adjuvant therapies for NENs eligible for PRRT. Clinical trials using such a personalized approach could evaluate agents that have been FDA-approved for use in other cancers. Especially compounds with preclinical evidence for radiosensitization effects together [^177^Lu]Lu-DOTA-TATE (marked with ◄) and those with evidence for anti-tumor effects in NENs (marked with #) should be prioritized.

Genetic or metabolic markers can guide selection of an appropriate combination therapy with agents that have been approved for other cancers (Figure 3). Tumors with transcriptional profiles, somatic or germline mutations indicating upregulation of the PI3K/AKT/mTOR might benefit from combinations with temsirolimus or one of the approved PI3K inhibitors. Such an approach might be effective in *RET*-mutated MTCs 173. On the other hand, PGLs with *SDHx* mutations or metabolic profiles indicating impairment of SDH or other Krebs cycle enzymes that are characterized by a CIMP might respond to combinations including HDAC or DNA methyltransferase inhibitors. PARP inhibitors might improve radiotherapeutic effects across a wider range of tumors, but should certainly be considered for tumors with mutations in DNA damage repair pathways, including *BRCA1/2*, *ATM*, and *ATRX*. Transcriptomics might further aid in predicting PARP resistance, including loss of TP53BP1 or Shieldin complex expression 174. Bioinformatic approaches might also assist in the selection of potential complementary therapies. The PanDrugs.org platform is one such example and provides suggestions for chemotherapeutics based on the tumor's mutational landscape 175.

Due to massive reductions in the costs of next generation sequencing, multi-gene panel genetic diagnostics are performed on a routine basis in the Western world. Especially for patients with PPGLs, genetic screening for mutations in known susceptibility genes is recommended to identify hereditary causes and to better manage the risk of metastatic disease 176. Additionally, several countries are running precision oncology-based cancer trials, including the National Institutes of Health in the USA and the Molecularly Aided Stratification for Tumor Eradication (MASTER) program in Germany. Personalized PRRT approaches could be incorporated in such existing infrastructures. The proposed combinational treatments might become important, especially, in the context of rare diseases, where dedicated clinical trials for new agents are difficult to perform.

It should be emphasized that evaluating the effectiveness of radionuclide therapies might require expanding the conventional approaches for measuring response. This expansion is necessary to adequately encompass the specific objectives and impacts of this therapeutic approach. Given the distinct mechanism and goals of radionuclide therapy, the objective response rates, such as tumor shrinkage, alone might not fully capture the actual clinical benefit for the patient. Hence, it's crucial to consider other factors, such as improvements in quality of life, reducing tumor burden, alleviating symptoms, slowing disease progression, or increased survival time, to gain a more comprehensive picture of treatment effectiveness. In this regard, the utilization of [^18^F]FDG-PET/CT in assessing therapy response can be a valuable complement to gain a more comprehensive understanding of treatment efficacy 177.

Currently running clinical trials that investigate combinational therapies with [^177^Lu]Lu-DOTA-TATE (Lutathera) target some of the pathways mentioned above, including DNA repair, epigenetics, and kinase/mTOR signaling (Table 1). The important point, however, is that although patients are selected based on SSTR2 positivity by [^68^Ga]Ga-DOTA-TATE PET/CT, they are not specifically chosen to receive inhibitors based on their tumor profile. In future clinical trials should incorporate personalized selection of complementary drugs also in the setting of PRRT.

Since [^177^Lu]Lu-DOTA-TATE itself was shown to have a mild toxicity profile 178, adverse effects are expected to originate from the second agent. Drug combinations with [^177^Lu]Lu-DOTA-TATE have so far resulted in little increase in toxicities, although the combination with everolimus had to be managed under close observation and dose reductions were necessary in some patients 153, 179. As the proposed strategy would address targets with high abundance in tumor tissue, lower doses of the second agent might be required, thereby limiting normal tissue toxicities.

## Conclusions and Outlook

Present systemic treatments offered to patients with NENs will halt disease progression in some individuals but rarely lead to a cure. Hence, further improvements and an increased level of personalization are urgently required. As radionuclide therapy shows promising responses in some patients and inhibitors of tumorigenic pathways were shown to be effective in certain patient groups, the next logical step is the application of combination therapies based on the knowledge of active tumorigenic pathways. The currently most promising strategies are targeting DNA repair pathways, which is applicable across a wide range of NENs, and inhibitors of kinase signaling that could be effective in subgroups of GEP-NENs, MTCs, and PPGLs. In this regard, clinical trials should incorporate adequate patient selection into their trial design. Genetic mutations, transcriptional or even microRNA signatures are feasible biomarkers. A third pathway with the potential for yielding improved treatments for some patients with SCLC, GEP-NEN, and PPGL is Hedgehog, a regulator of stemness features. In combination with other strategies, e.g. those directed towards dose maximization, personalized radiosensitization should become a mainstay of NEN therapy.

## Figures and Tables

**Table 1:** Clinical trials directed towards [^177^Lu]Lu-DOTA-TATE (Lutathera) in combination with other agents

Identifier	Phase	Conditions	Drugs	Category 2nd drug	Status
NCT05053854	I	metastatic GEP-NEN	Lutathera + Talazoparib	PARP inhibitor	recruiting
NCT04375267	I	NEN, ThymomaMesothelioma (SSTR2+)	Lutathera + Olaparib	PARP inhibitor	recruiting
NCT05870423	I	NEN	Lutathera + Olaparib	PARP inhibitor	recruiting
NCT04086485	I, II	GEP-NEN	Lutathera + Olaparib	PARP inhibitor	recruiting
NCT05724108	II	metastatic NENs	Lutathera + Triapine	ribonucleotide reductase inhibitor (DNA synthesis/repair)	recruiting
NCT04750954	I	NEN	Lutathera + Peposertib	DNA-dependent protein kinase inhibitor (DNA repair)	recruiting
NCT05178693	I	NEN	Lutathera + ASTX727	DNA hypomethylating agent	recruiting
NCT05687123	I	pancreatic NEN	Lutathera + Sunitinib	tyrosine kinase inhibitor	recruiting
NCT05249114	I	NEN	Lutathera + Carbozantinib	tyrosine kinase inhibitor	recruiting
NCT03629847	I, II	GEP- and lung NEN	Lutathera + Everolimus	mTOR inhibitor	unknown
NCT05142696	I	SCLC	Lutathera + Carboplatin+ Etoposide + Tislelizumab	alkylating agent, topoisomerase inhibitor, immunotherapy	recruiting
NCT02358356	II	GEP-NEN	Lutathera + Capecitabine + Temozolomide	antimetabolite, alkylating agent	completed
NCT03325816	I, II	SCLC	Lutathera + Nivolumab	immunotherapy	completed
NCT04525638	II	grade 3 neuroendocrine tumours,neuroendocrine carcinomas	Lutathera + Nivolumab	immunotherapy	recruiting
NCT03457948	II	NEN	Lutathera + Pembrolizumab	immunotherapy	recruiting
2014-003067-38*	II	GEP-NEN	Lutathera + Capecitabine	antimetabolite	ongoing

Sourced from ClinicalTrials.gov or *clinicaltrialsregister.eu
